# Anxiety Is Associated With Diverse Physical and Cognitive Symptoms in Youth Presenting to a Multidisciplinary Concussion Clinic

**DOI:** 10.3389/fneur.2021.811462

**Published:** 2022-02-07

**Authors:** Grant L. Iverson, Jonathan Greenberg, Nathan E. Cook

**Affiliations:** ^1^Department of Physical Medicine and Rehabilitation, Harvard Medical School, Boston, MA, United States; ^2^Department of Physical Medicine and Rehabilitation, Spaulding Rehabilitation Hospital, Boston, MA, United States; ^3^Spaulding Research Institute, Boston, MA, United States; ^4^MassGeneral Hospital for Children Sports Concussion Program, Boston, MA, United States; ^5^Department of Psychiatry, Integrated Brain Health Clinical and Research Program, Massachusetts General Hospital, Boston, MA, United States; ^6^Department of Psychiatry, Harvard Medical School, Boston, MA, United States

**Keywords:** anxiety, concussion, head injury, traumatic brain injury, sports, adolescents

## Abstract

**Introduction:**

Pre-injury and post-injury anxiety are prevalent and important to consider in the medical management of concussions in youth. We examined the association between anxiety and other physical, cognitive, and emotional symptoms in injured adolescents and young adults undergoing an initial evaluation in a specialty concussion clinic.

**Methods:**

Participants were 158 adolescents and young adults presenting to a multidisciplinary concussion clinic for evaluation and treatment (54.4% girls and women; mean age = 17.3 years; SD = 2.9). Their median days post injury was 29 (interquartile range = 14–49; range = 7–349). They were divided into binary groups based on whether they had a pre-injury history of anxiety diagnosis or treatment and whether they were experiencing current anxiety in the week prior to the evaluation, and then compared on the Post-Concussion Symptom Scale.

**Results:**

Youth with a pre-injury history of anxiety reported greater post-concussion symptoms (Md total score = 36.0, IQR = 21.5–53.0) compared to youth with no pre-injury history of anxiety (Md total score = 20.5, IQR = 6.0–36.0; MW U = 1,520.00 *p* = 0.001, *r* = 0.26, small-medium effect size). They reported significantly worse headaches, nausea, balance difficulty, dizziness, vision problems, fatigue, concentration difficulty, irritability, nervousness, sadness, feeling more emotional, trouble falling asleep, and sleeping more than usual. Youth with high post-injury anxiety reported greater post-concussion symptoms (Md total score = 55.0, IQR = 33.0–62.5) compared to youth with low post-injury anxiety (Md total score = 19.0, IQR = 6.0–35.0; MW U = 681.00, *p* < 0.001, *r* = 0.49, large effect size). They reported significantly worse headaches, nausea, vomiting, dizziness, vision problems, fatigue, sensitivity to light, feeling mentally foggy, feeling slowed down, concentration difficulty, memory difficulty, irritability, sadness, feeling more emotional, drowsiness, trouble falling asleep, sleeping less than usual, and sleeping more than usual. Logistic regressions revealed that both pre-injury and post-injury anxiety were strong predictors of persistent post-concussion symptoms, with high post-injury anxiety presenting the strongest independent predictor, while attention-deficit hyperactivity disorder and pre-injury migraines were not significant predictors. Essentially all adolescents with high post-injury anxiety (97.1%) and nearly 9 of 10 adolescents with pre-injury treatment for anxiety (87.8%) met criteria for persistent post-concussion symptoms.

**Discussion:**

Pre-injury and post-injury anxiety are important risk factors for greater post-concussion symptoms among adolescents and young adults. Elevated post-injury anxiety was the strongest predictor of persistent post-concussion symptoms. Assessment of anxiety is important among adolescents presenting for concussion care and delivery of evidence-supported treatments for anxiety are important considerations for treatment planning for these youth.

## Introduction

Concussions affect 42 million people worldwide annually (1). Psychological factors are important to consider when conceptualizing concussion symptomology and recovery (2–5). Anxiety, in particular, has the potential to amplify other symptoms, prolong recovery, and reduce quality of life following concussion. Core symptoms of generalized anxiety disorder, such as sleep disturbance, fatigue, irritability, and difficulty concentrating (6), are also core symptoms associated with prolonged recovery from concussion. A recent study of more than 37,000 adolescent student athletes undergoing baseline preseason testing indicated that more than eight out of 10 youth classified as having high anxiety (82.7%) met International Classification of Diseases and Related Health Problems-10th Revision (ICD-10) (7) symptom criteria for at least a mild form of the postconcussional syndrome compared to fewer than two out of 10 (18.4%) among those classified has having low anxiety, indicating that anxiety can mimic the post-concussion syndrome among youth that have not been injured (8).

Emerging evidence suggests that pre-injury anxiety plays a significant role in symptoms following concussion among youth and young adults. Adolescents with suspected acute concussions who had an anxious profile during the preseason reported much greater post-concussion symptoms within the first 72 h following injury than those without preseason anxiety (9). Moreover, anxiety sensitivity (beliefs that anxiety symptoms or arousal can have harmful consequences), is also associated with greater acute symptoms following concussion (10). The associations between pre-injury anxiety, or anxiety sensitivity, and amplified acute symptoms following concussion are particularly important because experiencing greater acute symptoms is a well-established risk factor for slower clinical recovery (5). Moreover, in a large-scale study of youth receiving care in concussion specialty clinics, pre-existing anxiety disorders were associated with more severe symptomology and prolonged recovery in children and adolescents (11). However, Kent et al. (12) reported that a pre-existing anxiety diagnosis was *not* associated with persistent symptoms after concussion, after adjusting for sex and a personal history of migraine, depression, and attention-deficit/hyperactivity disorder (ADHD). Thus, several, but not all, studies to date suggest that pre-injury anxiety is important to consider during the medical management of youth who sustain concussions.

Post-injury anxiety appears to be an important factor in recovery from concussion as well (13). Post-injury anxiety is associated with a slower recovery and slower return to sports participation among adolescents (14), and anxious symptom profiles are common among youth presenting to specialty concussion clinics (15). Preliminary evidence indicates that in such specialty clinics, anxiety rates are particularly high, with anxiety diagnoses evident in almost 60% of youth experiencing persistent symptoms and prolonged recovery in one study (16).

Concussion outcome research with youth generally follows two methodologies, one recruiting subjects who are acutely injured from schools, and usually medically monitored by athletic trainers, and the other recruiting subjects from specialty clinics. Research in specialty clinics is important because youth presenting for specialty care are at increased risk for slow recovery and persistent symptoms (11, 16–18). More research is needed from specialty clinics to better understand factors relating to persistent symptoms and, in particular, ways to advance and improve concussion treatment and rehabilitation.

The purpose of this study is to examine the association between anxiety and other physical, cognitive, and emotional symptoms in adolescents and young adults undergoing an initial evaluation in a specialty concussion clinic. We had three hypotheses. First, we hypothesized that youth who reported a personal history of anxiety diagnosis or treatment would endorse greater post-concussion symptoms at the time of their initial clinic visit. Second, we hypothesized that those with current post-injury anxiety would endorse substantially greater physical, cognitive, and emotional symptoms than youth who did not report current anxiety. Third, we hypothesized that current post-injury anxiety would be an independent predictor of persistent post-concussion symptoms after controlling for pre-injury vulnerabilities, such as ADHD, learning disorders, prior concussions, pre-injury treatment for migraines, and pre-injury anxiety diagnosis or treatment. It is important to consider and control for these pre-injury vulnerability factors because all of them are associated with greater baseline, pre-injury symptom reporting in adolescents (19, 20).

## Materials and Methods

### Participants

A retrospective cohort study of prospectively collected clinical data from 158 adolescents and young adults (ages 13–24), who presented for clinical care to the MassGeneral Hospital for Children Sports Concussion Program was conducted (see Table 1). Youth were included if they were age 13–24 and presented to care within 1 year of a concussion; youth were excluded if they sustained a moderate or severe traumatic brain injury. Individuals were seen for initial clinical evaluations a median (Md) of 29 days (IQR 14–49 days) after their concussion. Institutional review board approval to create and use this de-identified database was obtained from Mass General Brigham.

**Table 1:** Characteristics of the high and the low anxiety groups.

	**Pre-injury anxiety** **(***n*** = 41)**	**No pre-injury anxiety** **(***n*** = 112)**	**High post-injury anxiety** **(***n*** = 35)**	**Low post-injury anxiety** **(***n*** = 123)**
Age, M (SD)	17.6 (2.9)	17.2 (2.8)	18.1 (2.9)	17.1 (2.8)
**Gender**, ***n*** **(%)**				
Male	12 (29.3%)	58 (51.8%)	13 (37.1%)	59 (48.0%)
Female	29 (70.7%)	54 (48.2%)	22 (62.9%)	64 (52.0%)
**Pre-injury health**, ***n*** **(%)**				
Attention-deficit hyperactivity disorder	12 (29.3%)	13 (11.6%)	6 (17.6%)	19 (15.8%)
Learning disorder	9 (22.5%)	8 (7.1%)	4 (12.1%)	13 (10.8%)
Treatment for anxiety	41 (100%)	0 (0%)	15 (44.1%)	26 (21.8%)
Treatment for depression	18 (45.0%)	6 (5.4%)	9 (27.3%)	15 (12.5%)
Treatment for headaches	11 (26.8%)	21 (18.8%)	6 (17.6%)	26 (21.7%)
Treatment for migraines	7 (17.1%)	9 (8.0%)	4 (11.8%)	12 (10.0%)
**Previous concussions**, ***n*** **(%)**				
0	14 (34.1%)	48 (43.2%)	10 (28.6%)	54 (44.3%)
1	8 (19.5%)	22 (19.8%)	8 (22.9%)	23 (18.9%)
2 or more	19 (46.3%)	41 (36.9%)	17 (48.6%)	45 (36.9%)
Time since injury (days), mean, (SD)	54.6 (63.1)	49.2 (63.1)	74.9 (83.3)	42.5 (53.1)
Time since injury (days), median, (IQR)	32 (15.5–77.5)	27.5 (4–47)	36 (20–91)	27 (14–45)
Total symptom severity score, mean (SD)	37.8 (22.8)	25.4 (22.7)	51.9 (23.1)	22.3 (18.9)
Total symptom severity score, median, (IQR)	36 (21.5–53)	20.5 (6–36)	55 (33–62.5)	19 (6–35)

*Pre-injury health variables and prior concussion history had missing data for 1–5 subjects per variable*.

### Specialty Clinic

The MassGeneral Hospital for Children Sports Concussion Program is a multidisciplinary clinic providing individualized and holistic concussion treatment and rehabilitation, tailored to the specific problems and needs of the patient. The care team consists of specialists in rehabilitation medicine, clinical psychology, neuropsychology, and physical therapy. Patients typically undergo an initial evaluation with a rehabilitation medicine physician and based on the results of that evaluation, are referred to other members of the care team. For example, if a patient's initial evaluation indicates likely cervicogenic contributions to daily headaches as well as frequent anxiety, they might be prescribed medication to manage headaches and be referred for physical therapy and clinical psychology. Similarly, if the initial evaluation suggests dizziness and decreased concentration the patient might be referred to physical therapy for vestibular rehabilitation and neuropsychology to address cognitive symptoms. The clinic tends to treat patients with considerable complexity who are experiencing slow recovery and/or who present with complex health comorbidities.

### Measures and Definition of Anxiety

During the initial clinic visit, patients, often with the assistance of their caregivers, complete a standardized intake form that includes their self-reported health history. The intake form includes a question asking, “Have you been diagnosed with or received treatment for: Anxiety” (yes/no), and responses to this question were used to identify patients with a prior history of anxiety. In addition, patients complete the Post-Concussion Symptom Scale, PCSS (21, 22), a standardized self-report questionnaire that includes 22 symptoms (see Tables 2, 3) rated from zero to six in terms of severity, with 1 or 2 reflecting “mild,” 3 or 4 reflecting “moderate,” and 5 or 6 reflecting “severe” problems with a given symptom. Patients rated the PCSS items in reference to how they have been feeling over the last week before their clinic visit. The symptom ratings are summed to calculate a total score. The internal consistency of the scale ranges from 0.88 to 0.94 in high school and college students, and 0.92 to 0.93 in concussed athletes (22). One of the PCSS symptoms is “Nervousness,” and ratings of 3 or greater, reflecting ratings of “moderate” or “severe” problems over the last week, were used to characterize patients experiencing current post-injury anxiety.

**Table 2:** Endorsement of individual symptoms stratified by a personal history of treatment for anxiety.

	**Mild or greater symptoms**		**Moderate or greater symptoms**	
	**Pre-injury anxiety group (*n* = 41)**	**No pre-injury anxiety group (*n* = 112)**	**Pre-injury anxiety group (*n* = 41)**	**No pre-injury anxiety group (*n* = 112)**
Headache, *n* (%)	39 (95.1)	94 (83.9)	30 (73.2)	54 (48.6)
Vomiting, n (%)	5 (12.5)	6 (5.4)	3 (7.5)	3 (2.7)
Nausea, *n* (%)	23 (57.5)	32 (28.6)	13 (32.5)	12 (10.7)
Balance problems, *n* (%)	23 (57.5)	41 (36.9)	10 (25.0)	19 (17.0)
Dizziness, *n* (%)	31 (75.6)	56 (50.5)	14 (34.1)	21 (18.9)
Trouble falling asleep, *n* (%)	24 (60.0)	41 (36.6)	13 (32.5)	23 (20.7)
Fatigue, *n* (%)	30 (73.2)	68 (61.3)	20 (48.8)	34 (30.6)
Sleeping more than usual, *n* (%)	25 (64.1)	47 (42.0)	12 (30.8)	23 (20.5)
Sleeping less than usual, *n* (%)	12 (30.0)	20 (18.0)	7 (17.9)	12 (10.8)
Sensitivity to light, *n* (%)	32 (80.0)	71 (63.4)	16 (40.0)	37 (33.0)
Drowsiness, *n* (%)	25 (62.5)	56 (50.0)	14 (35.0)	24 (21.4)
Sensitivity to noise, *n* (%)	22 (55.0)	59 (53.6)	11 (27.5)	24 (21.6)
Irritability, *n* (%)	26 (65.0)	41 (36.6)	13 (32.5)	18 (16.1)
Nervousness, *n* (%)	25 (61.0)	38 (33.9)	15 (37.5)	19 (17.0)
Sadness, *n* (%)	23 (56.1)	27 (24.3)	11 (26.8)	16 (14.4)
Feeling more emotional, *n* (%)	23 (56.1)	32 (28.8)	11 (26.8)	16 (14.4)
Numbness or tingling, *n* (%)	6 (15.4)	13 (11.9)	2 (5.1)	2 (1.8)
Feeling mentally foggy, *n* (%)	33 (82.5)	72 (64.9)	16 (41.0)	34 (30.6)
Feeling slowed down, *n* (%)	29 (70.7)	65 (58.6)	16 (40.0)	37 (33.3)
Difficulty concentrating, *n* (%)	34 (85.0)	78 (69.6)	22 (55.0)	41 (36.6)
Difficulty remembering, *n* (%)	26 (65.0)	61 (54.5)	15 (37.5)	27 (24.1)
Visual problems, *n* (%)	24 (58.5)	40 (36.7)	10 (24.4)	10 (9.3)

**Table 3:** Endorsement of individual symptoms stratified by current anxiety.

	**Mild or greater symptoms**		**Moderate or greater symptoms**	
	**High post-injury anxiety group** **(***n*** = 35)**	**Low post-injury anxiety group** **(***n*** = 123)**	**High post-injury anxiety group** **(***n*** = 35)**	**Low post-injury anxiety group** **(***n*** = 123)**
Headache, *n* (%)	33 (94.3)	105 (85.4)	27 (77.1)	60 (49.2)
Vomiting, *n* (%)	5 (15.2)	6 (4.9)	3 (9.1)	3 (2.4)
Nausea, *n* (%)	18 (51.4)	6 (4.9)	12 (34.3)	15 (12.3)
Balance problems, *n* (%)	14 (41.2)	50 (41.3)	11 (32.4)	18 (14.8)
Dizziness, *n* (%)	25 (73.5)	65 (52.8)	16 (47.1)	21 (17.1)
Trouble falling asleep, *n* (%)	21 (61.8)	47 (38.2)	17 (50.0)	20 (16.4)
Fatigue, *n* (%)	28 (82.4)	74 (60.2)	20 (58.8)	36 (29.3)
Sleeping more than usual, *n* (%)	22 (64.7)	52 (42.6)	12 (35.3)	25 (20.5)
Sleeping less than usual, *n* (%)	11 (32.4)	22 (18.0)	10 (29.4)	10 (8.3)
Sensitivity to light, *n* (%)	29 (85.3)	78 (63.4)	21 (61.8)	34 (27.6)
Drowsiness, *n* (%)	22 (64.7)	61 (50.0)	18 (52.9)	21 (17.2)
Sensitivity to noise, *n* (%)	21 (63.6)	62 (50.8)	13 (39.4)	24 (19.5)
Irritability, *n* (%)	27 (79.4)	43 (35.0)	21 (61.8)	12 (9.8)
Nervousness, *n* (%)	35 (100)	30 (24.4)	35 (100)	0 (0)
Sadness, *n* (%)	26 (74.3)	26 (21.3)	19 (54.3)	8 (6.6)
Feeling more emotional, *n* (%)	29 (82.9)	28 (23.1)	19 (54.3)	10 (8.3)
Numbness or tingling, *n* (%)	6 (18.8)	13 (10.7)	2 (6.3)	2 (1.7)
Feeling mentally foggy, *n* (%)	31 (91.2)	78 (63.9)	23 (69.7)	30 (24.6)
Feeling slowed down, *n* (%)	32 (91.4)	65 (53.3)	22 (64.7)	33 (27.0)
Difficulty concentrating, *n* (%)	31 (88.6)	84 (68.9)	23 (67.6)	42 (34.1)
Difficulty remembering, *n* (%)	25 (73.5)	65 (52.8)	19 (55.9)	25 (20.3)
Visual problems, *n* (%)	22 (66.7)	45 (36.9)	11 (33.3)	9 (7.4)

*To be included in the high post-injury anxiety group, participants had a score of 3 or greater on the nervousness item. As such, 100% of participants in the high anxiety group have indicated the presence of nervousness as a symptom*.

### ICD-10 Postconcussional Syndrome

We also dichotomized the PCSS symptoms into mild and moderate postconcussional syndrome based on the ICD-10 symptom criteria. To meet the ICD-10 postconcussional syndrome criteria, participants had to endorse at least 1 symptom in at least 3 of the following categories: (i) physical (headache, nausea, balance problems, dizziness, fatigue, sensitivity to noise, and sensitivity to light), (ii) cognitive (difficulty concentrating, difficulty remembering, and feeling mentally foggy), (iii) emotional (irritability, sadness, and feeling more emotional), and (iv) insomnia (trouble falling asleep or sleeping less than usual). Of note, responses on “nervousness” were not used for constructing the postconcussional syndrome variable. For mild postconcussional syndrome, ratings of mild severity (≥1) were considered as symptom endorsement. Ratings of 3 or greater for at least 1 symptom in at least 3 categories were considered as symptom endorsement sufficient for a moderate postconcussional syndrome classification.

### Statistical Analyses

Due to non-normally distributed data, Mann-Whitney U tests were conducted to compare groups (Pre-Injury Anxiety vs. No Pre-Injury Anxiety; High Current Anxiety vs. Low Current Anxiety), on symptom ratings, including the PCSS total score and individual item ratings. Low current anxiety was defined as a nervousness score of 0–2 and high current anxiety was defined as a nervousness score of 3–6. The Z values from the Mann-Whitney U test were used to calculate non-parametric effect sizes (*r* = $\frac{z}{\sqrt{}N}$) (23), which were interpreted according to conventional guidelines, i.e., *r* = 0.1, small; *r* = 0.3, medium; *r* = 0.5, large (24). Logistic regression was used to determine which covariates were associated with meeting criteria for mild postconcussional syndrome as the dichotomous outcome variable to maximize the ratio of participants with each outcome category to the number of covariates. For the first model, the following variables were included as covariates: gender (male = 0; female = 1), ADHD (no = 0; yes = 1), migraines (no = 0; yes = 1), prior concussions (0 prior concussions = 0; 1 or more prior concussions = 1), and prior treatment for anxiety (no = 0; yes = 1). For the second model, all these covariates were also included with the addition of current anxiety (low current anxiety = 0; high current anxiety = 1). All covariates were entered in a single step for each model.

## Results

A total of 158 participants were included in this study. The mean age of participants was 17.3 years (SD = 2.9; range 13–24). The sample included modestly more girls/young women (54.4%) than boys/young men (45.6%). Racial and ethnic information about the participants was not available. The majority of participants (63.7%) presented for care following a concussion sustained during sports or recreational activities. Roughly 1 in 5 patients (19.4%) reported experiencing brief loss of consciousness and about 1 in 4 (27.9%) reported some degree of post-traumatic amnesia following their injury. The proportions of adolescents with pre-injury treatment for anxiety (20.0%) and without pre-injury treatment for anxiety (19.1%) who reported loss of consciousness at the time of injury did not differ, $\chi_{\left(1\right)}^{2}$ = 0.02, *p* = 0.90. However, a greater proportion of adolescents with pre-injury treatment for anxiety (40.0%) reported posttraumatic amnesia compared to those without pre-injury treatment for anxiety (22.7%), $\chi_{\left(1\right)}^{2}$ = 4.41, *p* = 0.04. Demographic and health history information about the participants is summarized in Table 1. Significantly more adolescents with pre-injury anxiety reported a pre-injury history of depression (45.0%) compared to adolescents without pre-injury anxiety (5.4%), $\chi_{\left(1\right)}^{2}$ = 34.84, *p* < 0.001. To account for this potential confound, we conducted sensitivity analyses, repeating the primary statistical analyses after excluding those with a pre-injury history of depression (*n* = 24) and the primary findings reported below did not change.

### Pre-injury Treatment for Anxiety

Adolescents and young adults with a pre-injury history of anxiety reported greater post-concussion symptoms (Md total score = 36.0, IQR = 21.5–53.0) compared to youth with no pre-injury history of anxiety (Md total score = 20.5, IQR = 6.0–36.0; MW U = 1,520.0, *p* = 0.001, *r* = 0.26, small to medium effect size). They reported significantly worse headaches (MW U = 1,699.0, *p* = 0.01, *r* = 0.20), nausea (MW U = 1,526.0, *p* = 0.001, *r* = 0.28), balance difficulty (MW U = 1,725.0, *p* = 0.02, *r* = 0.18), dizziness (MW U = 1,552.0, *p* = 0.002, *r* = 0.26), vision problems (MW U = 1,658.5, *p* = 0.007, *r* = 0.22), fatigue (MW U = 1,769.5, *p* = 0.03, *r* = 0.18), concentration difficulty (MW U = 1,723.5, *p* = 0.02, *r* = 0.19), irritability (MW U = 1,594.5, *p* = 0.003, *r* = 0.24), nervousness (MW U = 1,620.0, *p* = 0.002, *r* = 0.25), sadness (MW U = 1,608.0, *p* = 0.001, *r* = 0.27), feeling more emotional (MW U = 1,669.5, *p* = 0.003, *r* = 0.24), trouble falling asleep (MW U = 1,771.0, *p* = 0.03, *r* = 0.18), and sleeping more than usual (MW U = 1,700.5, *p* = 0.03, *r* = 0.18). The groups did not differ in age or time since injury. However, the group with pre-injury treatment for anxiety included significantly more girls/young women (70.7%) compared to the group with no pre-injury treatment for anxiety (48.2%), $\chi_{\left(1\right)}^{2}$ = 6.13, *p* = 0.01.

### Post-injury Anxiety

Youth with high post-injury nervousness reported greater post-concussion symptoms compared to youth with low post-injury nervousness (MW U = 681.0, *p* < 0.001, *r* = 0.49, large effect size). Those with high post-injury nervousness (Md = 36 days, IQR 14-45) presented to the clinic later compared to youth with low post-injury nervousness (Md = 27 days, IQR 20-91; MW U = 1,554.53, *p* = 0.01, *r* = 0.20, small effect size). The groups did not differ in gender or age. Slightly more than half of the adolescents who experienced high post-injury nervousness (55.9%) did not report a pre-injury history of anxiety treatment.

Regarding individual symptoms, youth with high post-injury nervousness reported significantly worse headaches (MW U = 1,375.0, *p* = 0.001, *r* = 0.26), nausea (MW U = 1.608.5, *p* = 0.01, *r* = 0.21), vomiting (MW U = 1,823.0, *p* = 0.04, *r* = 0.16), dizziness (MW U = 1,428.0 *p* = 0.003, *r* = 0.24), vision problems (MW U = 1,186.5 *p* < 0.001, *r* = 0.32), fatigue (MW U = 1,305.5, *p* = 0.001, *r* = 0.27), sensitivity to light (MW U = 1,267.0, *p* < 0.001, *r* = 0.29), feeling mentally foggy (MW U = 946.0, *p* < 0.001, *r* = 0.40), feeling slowed down (MW U = 1,020.0, *p* < 0.001, *r* = 0.39), concentration difficulty (MW U = 1,197.5, *p* < 0.001, *r* = 0.32), memory difficulty (MW U = 1,311.5, *p* = 0.001, *r* = 0.28), irritability (MW U = 845.0, *p* < 0.001, *r* = 0.47), sadness (MW U = 799.0, *p* < 0.001, *r* = 0.54), feeling more emotional (MW U = 673.5, *p* < 0.001, *r* = 0.57), drowsiness (MW U = 1,439.0, *p* = 0.004, *r* = 0.23), trouble falling asleep (MW U = 1,358.0, *p* = 0.001, *r* = 0.28), sleeping less than usual (MW U = 1,706.5, *p* = 0.03, *r* = 0.18), and sleeping more than usual (MW U = 1,468.5 *p* = 0.005, *r* = 0.23).

### Predicting ICD-10 Postconcussional Syndrome

In the first model, five predictor variables (listed in Table 4) were entered in a logistic regression with mild postconcussional syndrome classification as the dichotomous outcome. The logistic regression model was statistically significant (*p* = 0.001) and well-calibrated (Hosmer and Lemeshow, *p* = 0.68). Measures of discrimination suggested modest prediction accuracy [Nagelkerke *R*^2^ = 0.18; area under the receiver operating curve (AUC) = 0.71, *p* < 0.001]. As seen in Table 4, female gender and prior treatment for anxiety had a significant relationship with postconcussional syndrome classification, with prior treatment for anxiety associated with 5 times greater odds of meeting criteria for postconcussional syndrome.

**Table 4:** Logistic regression results and base rates of symptom reporting similar to mild ICD-10 postconcussional syndrome youth with anxiety.

	**Model 1**	**Model 2**	
	**Odds ratio (CI)**	**Odds ratio (CI)**	**% with mild ICD-10 postconcussional syndrome**
Gender	2.26 (1.03–4.99)*	2.39 (1.05–5.44)*	Girls/young women: 76.7% Boys/young men: 52.8%
ADHD	0.53 (0.19–1.52)	0.46 (0.15–1.48)	ADHD: 60.0% No ADHD: 67.4%
Migraines	1.12 (0.27–4.65)	1.07 (0.24–4.79)	Migraines: 81.3% No migraines: 64.5%
Prior concussions	1.60 (0.74–3.41)	1.24 (0.55–2.78)	0: 63.5% 1+: 67.7%
Prior Tx for anxiety	4.93 (1.66–14.70)**	4.92 (1.51–16.03)**	Prior anxiety tx hx: 87.8% No prior anxiety tx hx: 58.0%
High current nervousness	(excluded)	24.12 (3.02–192.76)**	High current anxiety present: 97.1% High current anxiety absent: 56.9%

CI, 95% confidence interval; ADHD, Attention Deficit Hyperactivity Disorder; Tx/tx, Treatment; *signifies statistical significance at the 0.05 level, **signifies significance at the 0.01 level. Symptom ratings were derived from the Post-Concussion Symptom Scale, a dimensional scale that includes 22 items that are rated from zero to six, with 1–2 being mild, 3–4 being moderate, and 5–6 being severe. To meet postconcussional syndrome diagnostic criteria, the youth must endorse one or more symptoms in 3 out of 4 symptom domains: Physical Symptoms (headache, dizziness, balance problem, noise sensitivity, light sensitivity, and/or fatigue), Emotional Symptoms (irritability, sadness, and/or feeling more emotional), Cognitive Symptoms (poor concentration, poor memory, and/or feeling mentally foggy), and Insomnia (trouble falling asleep and/or sleeping less than usual). The symptom domain must have been endorsed as simply “present” (i.e., mild or greater symptoms). We excluded the symptom “nervousness” in the coding of postconcussional syndrome status because it was used as a predictor.

In the second model, a sixth predictor, high current anxiety, was added. The second logistic regression model was also statistically significant (*p* < 0.001) and well-calibrated (Hosmer and Lemeshow, *p* = 0.96). Measures of discrimination suggested modest prediction accuracy [Nagelkerke *R*^2^ = 0.33; area under the receiver operating curve (AUC) = 0.78, *p* < 0.001]. The significant predictors (in descending order based on odds ratios) were high current nervousness, prior treatment for anxiety, and female gender. Patients with high current anxiety had 24 times greater odds of meeting criteria for postconcussional syndrome.

## Discussion

This study examined the association between anxiety and other physical, cognitive, and emotional symptoms in adolescents and young adults undergoing an initial evaluation in a specialty concussion clinic. We first hypothesized that patients who reported a personal history of treatment for anxiety would endorse greater post-concussion symptoms at the time of their initial clinic visit. This hypothesis was supported, as participants with a prior history of treatment for anxiety reported significantly greater physical symptoms, such as headaches, nausea, balance difficulty, dizziness, vision problems, fatigue, trouble falling asleep, and sleeping more than usual compared to those without a history of treatment for anxiety. They also reported greater emotional symptoms, such as irritability, nervousness, sadness, and feeling more emotional. These results are consistent with prior studies illustrating that pre-injury anxiety is associated with reporting greater post-injury physical, cognitive, and emotional symptoms (11, 25, 26), and point to pre-injury anxiety as an important risk factor for greater post-concussion symptoms.

Our second hypothesis, that adolescents and young adults with current post-injury anxiety (i.e., operationalized as endorsing “nervousness” as “moderate” or “severe”) would endorse substantially greater physical, cognitive, and emotional symptoms than those who did not report current anxiety, was also supported. Post-injury nervousness, reported at the time of their first clinic visit, was strongly associated with experiencing greater post-injury physical (e.g., headaches, nausea, vomiting, dizziness, vision problems, fatigue, sensitivity to light, sleep disturbance), cognitive (feeling mentally foggy, concentration difficulty, and memory difficulty) and emotional (e.g., irritability, sadness, and feeling more emotional) symptoms. These results are consistent with prior studies suggesting that post-injury anxiety is common (16), associated with greater overall symptom reporting (27, 28), and is a risk for slower recovery (14).

Interestingly, those with high post-injury anxiety (i.e., “nervousness”) presented, on average, later to the specialty concussion clinic. Although we are unable to determine the reason for this, one possibility is that individuals with higher levels of anxiety were more prone to avoidance-based coping (29), which may have led to a longer period before seeking treatment.

Our third hypothesis, that the association between post-injury anxiety and post-concussion symptoms would remain after controlling for pre-injury vulnerabilities that are known to be associated with greater symptom reporting in youth, especially ADHD (19, 30) and pre-injury treatment for migraines (19, 31), was supported. Youth with ADHD (19, 30) or a personal history of migraine (19, 31) report diverse physical, cognitive, and emotional symptoms at baseline, during preseason testing—more so than youth who do not have these conditions. ADHD and pre-injury migraine were *not* significantly associated with post-injury symptom reporting after controlling for pre-injury anxiety in this clinic sample (Model 1, Table 4). It is also interesting to note that the association between post-concussion symptoms and pre-injury anxiety was not attenuated after putting *post-injury anxiety* in the model (Model 2, Table 4), indicating that a personal pre-injury history of anxiety is still associated with post-injury symptom reporting after controlling for elevated levels of post-injury anxiety.

There are important clinical implications for the finding that anxiety, and especially elevated post-injury nervousness, has such a strong association with other symptoms and problems following concussion. Essentially all adolescents with high post-injury anxiety (97.1%) and nearly 9 of 10 adolescents with pre-injury treatment for anxiety (87.8%) met criteria for postconcussional syndrome. Meta-analytic evidence convincingly demonstrates that psychotherapy, namely cognitive behavior therapy (CBT), is effective for treating anxiety among adolescents (32, 33). Assessment of anxiety is important among adolescents presenting for concussion care and could include brief self- or caregiver report screening questionnaires, focused inquiry during clinical interview, or even reviewing responses to the “nervousness” or similar items on a concussion symptom checklist. Moreover, when heightened anxiety is identified or suspected, referrals for evidence-supported treatments for anxiety, such as CBT, are important considerations for treatment planning for these youth.

Taken together, results indicate that both pre-injury anxiety diagnosis or treatment and post-injury anxiety are important prognostic variables, and contribute substantially to post-concussion symptoms. This suggests that treatment for anxiety might dampen or ameliorate diverse physical, cognitive, and emotional symptoms in those who are slow to recover from concussion.

### Limitations

Several limitations of this study should be considered. First, the collected information about pre-injury anxiety diagnosis or treatment was limited, and did not include data on the onset, diagnosis, severity, duration, or type of treatment. Although the existence of pre-injury treatment for anxiety may be taken as evidence for clinically significant anxiety symptoms, the nature and severity of pre-injury anxiety was not possible to quantify, and it is possible that some may have experienced pre-injury anxiety without a formal diagnosis or prior treatment. Second, two of our hypotheses were based on post-injury ratings of nervousness. Although nervousness and anxiety are sometimes used interchangeably (34), nervousness does not imply the existence of an anxiety disorder. Third, given the retrospective design of our study, we were limited in terms of the potential pre-injury differences that may exist between these groups and able to analyze only the select health history variables routinely collected during clinic intake. Finally, all data collected in this study were based on self-report, which inherently carry the risk of bias. More specifically, we compared adolescents on their total PCSS symptom scores based on their responses on one of the PCSS items (high vs. lower current nervousness), which introduces the possibility of a negative response bias, such that those endorsing high current nervousness might be prone toward endorsing many other PCSS items at the higher end of the response scale. Future research could incorporate a specialized measure of response bias or symptom validity to further examine the association found in our study. Nevertheless, findings of this study fit well with the majority of existing literature in the field, providing further support for the data and their interpretation. Future studies with more detailed and explicit measures of anxiety and its treatment may further establish and solidify the specific role played by anxiety in post-concussion symptoms among adolescents and young adults.

### Conclusions

Pre-injury treatment for anxiety and post-treatment anxiety are important prognostic risk factors for greater post-concussion symptoms among adolescents and young adults with concussion, even when taking into account common vulnerability factors. Post-injury anxiety is especially important and strongly associated with a diverse range of other symptoms. Assessment of pre-injury and post-injury anxiety may be important for the prognosis and treatment plans following concussion. More research is needed on how to customize treatment and rehabilitation for these adolescents and young adults, and to evaluate whether those interventions promote a more rapid and durable clinical recovery.

## Data Availability Statement

The original contributions presented in the study are included in the article, further inquiries can be directed to the corresponding author. The statistical code, syntax, output, and analyses are available to qualified researchers upon request. Requests should be directed to Grant L. Iverson, giverson@mgh.harvard.edu.

## Ethics Statement

The studies involving human participants were reviewed and approved by Mass General Brigham. Written informed consent from the participants' legal guardian/next of kin was not required to participate in this study in accordance with the national legislation and the institutional requirements.

## Author Contributions

GI conceptualized the study, completed the literature review, wrote sections of the manuscript, helped design the database, supervised the construction of the database, conceptualized the statistical analyses, provided internal funding for the study, and agrees to be accountable for the content of the work. JG assisted with reviewing literature, wrote sections of the manuscript, and agrees to be accountable for the content of the work. NC helped conceptualize the study, helped design and supervise the construction of the database, assisted with conceptualizing and running the statistical analyses, and wrote sections of the manuscript, and agrees to be accountable for the content of the work. All authors contributed to the article and approved the submitted version.

## Funding

GI acknowledges unrestricted philanthropic support from the Mooney-Reed Charitable Foundation, the National Rugby League, ImPACT Applications, Inc., Boston Bolts, and the Spaulding Research Institute. These entities were not involved in the study design, collection, analysis, interpretation of data, the writing of this article or the decision to submit it for publication. JG's contribution was supported by a grant from the National Center for Complementary and Integrative Health (1K23AT01065301A1).

## Conflict of Interest

GI serves as a scientific advisor for NanoDX^®^, Sway Operations, LLC, and Highmark, Inc. He has a clinical and consulting practice in forensic neuropsychology, including expert testimony, involving individuals who have sustained mild TBIs (including former athletes), and on the topic of suicide. He has received research funding from several test publishing companies, including ImPACT Applications, Inc., CNS Vital Signs, and Psychological Assessment Resources (PAR, Inc.). He has received research funding as a principal investigator from the National Football League, and subcontract grant funding as a collaborator from the Harvard Integrated Program to Protect and Improve the Health of National Football League Players Association Members. The remaining authors declare that the research was conducted in the absence of any commercial or financial relationships that could be construed as a potential conflict of interest.

## Publisher's Note

All claims expressed in this article are solely those of the authors and do not necessarily represent those of their affiliated organizations, or those of the publisher, the editors and the reviewers. Any product that may be evaluated in this article, or claim that may be made by its manufacturer, is not guaranteed or endorsed by the publisher.
